# Transcriptome Comparison of Chorion-Attached and Non-chorion-attached Endometrium in Mid-gestation of Rabbit

**DOI:** 10.3389/fvets.2022.838802

**Published:** 2022-03-10

**Authors:** Xiuli Mei, Ling Xu, Yan Ren, Minjie Yu, Liangde Kuang, Congyan Li, Yan Zhang, Chuanzhi Lu, Zhicheng Wang, Zhiqiang Guo, Xiaohong Xie, Dengping Huang, Ming Zhang

**Affiliations:** ^1^Animal Breeding and Genetics Key Laboratory of Sichuan Province, Sichuan Animal Science Academy, Chengdu, China; ^2^College of Animal Science and Technology, Sichuan Agricultural University, Chengdu, China; ^3^Farm Animal Genetic Resources Exploration and Innovation Key Laboratory of Sichuan Province, Sichuan Agricultural University, Chengdu, China

**Keywords:** endometrium, gene expression profiles, immune regulation, molecular adhesion, chorion

## Abstract

**Background:**

The chorion from the placenta is directly attached to the endometrium (CA) after embryo implantation while some parts of the endometrium are not chorion-attached (NCA). The differences in gene expression between the CA and NCA endometrium mid-gestation are unknown. Our objective was to compare the gene expression profiles of the CA and NCA endometrium of rabbit, to identify the differentially expressed genes (DEGs), and correlate the differences with the physiological state of the endometrium at mid-gestation of rabbit.

**Methods:**

We used transcriptome sequencing to reveal the differences in gene expression between CA and NCA endometrium (*n* = 3), and then determined the concentration of inflammatory cytokines in CA and NCA tissue and serum by ELISA.

**Results:**

Six Hundred and Forty-Six DEGs were identified between the CA and NCA endometrium [*p* < 0.05, |log2 (fold change) |≥ 2], The expression levels of 590 DEGs were higher in the NCA endometrium than in the CA endometrium, while the expression level of only 56 DEGs were higher in CA than in NCA. The DEGs were enriched in gene ontology (GO) terms and pathways related to immune regulation and cellular adhesions. Six hub-genes related to inflammatory mediator regulation of transient receptor potential (TRP) channels and chemokine signaling pathways had a lower expression level in the CA endometrium compared to the NCA endometrium, and the expression levels of genes related to focal adhesion and extracellular matrix (ECM)-receptors were significantly higher in NCA endometrium than in CA endometrium. The level of pro-inflammatory cytokines accumulated in the CA endometrium, and high abundance of integrin-β and THBS1 were localized in the luminal epithelium of the NCA endometrium, but not in the CA endometrium.

**Conclusions:**

Our study reveals differences in gene expression between the CA and NCA endometrium at mid-gestation of rabbit, and suggests implications for endometrial physiological function. The CA endometrium showed relative low-level gene expression compared to the NCA endometrium, while the NCA endometrium performed physiological functions related to focal adhesion and ECM-receptor interaction.

## Introduction

In the process of pregnancy maintenance, the dialogue at the maternal-fetal interface includes not only direct mediator transfer in the chorion-attached (CA) region between chorion and endometrium, but also an endocrine response in the non-chorion-attached (NCA) endometrial area (1–3). The endometrium undergoes morphological and functional changes at the implantation site, allowing for invasion of fetal trophoblasts and conjugation between the endometrium and the chorionic membranes (4). The change in endometrial morphology and function is called decidualization and decidua formation is critical for blastocyst implantation (1, 2). Endometrial decidualization under progesterone-induction allows receptivity of the embryo (2, 4). The decidualized endometrium can be divided into two areas, the CA and NCA region after embryo implantation. The cross-talk between mother and fetus depends on direct tissue communication between the endometrium and chorionic membrane, or through the hormones and cytokines by paracrine secretion (4–6). The endometrium provides a special milieu and the spatial expression of endometrial genes plays an important role in the maintenance of pregnancy (7). Therefore, we speculated that there might be differences in gene expression between the CA and NCA endometrium; however, few studies have focused on the differences in physiological function of CA and NCA endometria after embryo implantation.

The rabbit has a hemochorial discoid placenta similar to a human, and the hemochorial discoid placenta is comprised of trophoblast cells, mesenchymal cells, vasculature and maternal endometrium (8). The fetal chorionic trophoblast cells invade the maternal uterine arteries and veins at the maternal-fetal interface for controlling the bidirectional flow of nutrients and metabolites (9, 10), and the trophoblast cell population contributes to the endocrine activities of the placenta (11, 12). The decidualized endometrium has a crucial role in the process of pregnancy as the maternal tissue that first comes into direct contact with the embryo and allows for its proper implantation, survival, and development (13). Over the past few decades, extensive investigations have been carried out to clarify the molecular mechanisms of endometrial decidualization and receptivity, and embryo attachment, implantation, and trophoblast invasion (13–15). Moreover, some studies had revealed that the trophoblast cells invading the decidualized endometrium experienced the strongest immune challenge from the maternal immune system (16), but the allogeneic placental trophoblast and fetus are not attacked by the maternal immune system (17, 18). Some specifically expressed genes: (*Tpbp, Plac1, Syncytin*, and the retrotransposon-associated genes, *Peg10, Rtl1*, Endothelin B receptor, *Insl4, Leptin, Midline1*, and Pleiotrophin, *Gcm1, Mash2, Rhox, Esx1, Cathepsin, PAG, TKDP, Psg* and *Siglec*), the enhancer elements (glycoprotein hormone α-subunit) and gene isoforms (*3*β*HSD, Cyp19*) have been identified at the maternal-fetal interface of human and mouse (18–20). The fetal rabbit at mid-gestation is increasingly being used as a model for diagnosis of fetal abnormalities and disease treatment (21). It is a critical point in pregnancy with the transformation of progestogenic maintenance of pregnancy from corpus luteum dependence to placental dependence (22). However, the spatial expression of specific genes in the CA and NCA endometrium at mid-gestation after implantation still remains unknown.

Many studies of the placental transcriptome have provided us with a basic understanding of how the immunoregulation and adhesion function of the early placenta support embryo implantation and immune evasion (1, 2). The differences in physiological function between the CA and NCA endometrium at mid-gestation were not considered, however. The specific immune milieu that is provided by the CA and the endometrium NCA together is important for maintenance of pregnancy (23). Knowledge about the gene expression profile difference in the CA and NCA endometrium at mid-gestation of rabbit will provide a better understanding of the physiological functions of the endometrium in different regions, as well as how dysregulation of maternal immune tolerance can cause pregnancy failure at mid-gestation.

In this study we determined the transcriptome of chorion-attached and non-chorion-attached endometrium at mid-gestation of female rabbits using second-generation sequencing. We compared the genes specifically expressed in CA and NCA endometrium to elucidate the main physical functions of the maternal endometrium at mid-gestational stage.

## Materials and Methods

### Animals and Experiment Design

Three sexually mature and primiparous female *New Zealand* white rabbits (2.50 ± 0.15 kg, 6-moa) were performed estrus synchronization. Each rabbit was intravenously injected with 80 IU of human chorionic gonadotropin (hCG) at 48 h after being injected intravenously with 120 IU of equine chorionic gonadotropin (eCG) followed by natural mating. Diagnosis of pregnancy was performed on the 10th day after natural mating by abdominal palpation. On the 14th day of gestation, endometrial tissue in placental area were collected to isolate chorion-attached endometrium, and endometrium in non-placental area were also collected (Supplementary Figure 1). These tissues were immediately put into RNAlater™ stabilization solution (Thermo-Fisher, USA). The chorio-allantoic membranes and the chorion were carefully removed from endometrial tissue in placental area under a stereomicroscope (Leica MZ75, Leica Microsystems, Germany), and then only endometrium was collected as chorion-attached (CA) endometrium. Endometrial tissue in non-placental area was as non-chorion-attached (NCA) endometrium. Three CA and NCA endometrial tissues were collected from one individual and then, respectively mixed into CA and NCA sample pool. Finally, the sample tissue was divided into CA and NCA endometrium, and RNA-sequencing was performed using three individuals (*n* = 3). Collected tissue samples for ELISA were frozen at −80°C. CA endometrium for hematoxylin-eosin (H&E) staining and immunostaining were immediately fixed in 10% formalin PBS, and NCA endometrium samples were embedded in O.C.T compound for cryosectioning. Sampling accuracy was evaluated by microstructure of H&E staining (Supplementary Figure 1). Transcriptomics analysis was performed by Jingjie PTM Biolab Ltd. Co. (Hangzhou, China).

All animals were housed in individual cages under a 14-h light and 10-h dark regimen at a temperature of 16–25°C, and fed *ad libitum* on a standard diet (Laboratory Animal Nutrients, rabbit feed, GB 14924.3-2010, China). All experimental procedures were approved by the Animal Ethics Monitoring Committee of Sichuan Agricultural University (Appr. No. SASA201905) and carried out in accordance with the Guidelines of Animal Welfare in China.

### Total RNA Extraction

Total RNA from the CA and NCA tissues were extracted with TRIzol reagent (Invitrogen, CA, USA) and purified with a RNeasy column (Qiagen, USA) according to the manufacturer's protocol. RNA purity and integrity were assessed using the Nano Photometer® spectrophotometer (Bio-Rad, CA, USA) and the RNA Nano 6000 assay kit of the Bioanalyzer 2100 system (Agilent Technologies, CA, USA), respectively. RNA concentration was measured using Qubit® RNA assay kit in a Qubit® 2.0 Fluorometer (Life Technologies, CA, USA).

### Messenger RNA Library Construction and Sequencing

Only qualified RNA samples (RIN >8, OD_260/280_ = 1.9-2.2 28S:18S ≥ 1.5) were used for library construction and qualified libraries were used for sequencing. Sequencing libraries were generated using NEBNext® Ultra™ RNA Library Prep Kit for Illumina^®^ (NEB, USA) following manufacturer's protocol, and index codes were added to attribute sequences to each sample. Briefly, mRNA was purified from 5 μg of total RNA using poly-T oligo-conjugated magnetic beads. Following purification, the mRNA was fragmented into small pieces using divalent cations under elevated temperature in NEBNext first-strand synthesis reaction buffer, and the cleaved RNA fragments were reverse-transcribed into the final complementary DNA (cDNA) library according to the NEBNext^®^ Ultra™ RNA library prep kit protocol (NEB, USA). After adenylation of 3' ends of DNA fragments, the NEBNext adaptor with hairpin loop structure was ligated to prepare for hybridization. Fragments of cDNA, 150–200 bp in length, were purified by the AMPure XP system (Beckman Coulter, Beverly, USA) and library quality was assessed on the Agilent Bioanalyzer 2100 system using Qubit2.0 software. Lastly, the RNA libraries were sequenced on an Illumina HiSeq2000 platform, and the raw data were deposited in Gene Expression Omnibus (GEO) Datasets on NCBI (GSE152905 https://www.ncbi.nlm.nih.gov/geo/query/acc.cgi?acc=GSE152905).

### Transcriptome Data Analysis

Clean data were obtained by removing reads containing adapters, reads containing >10% poly-N and low-quality reads (>50% of bases had Phred quality score ≤ 10) from raw data. At the same time, Q20, Q30 and GC content in the clean data were calculated. All subsequent analyses were based on clean, high-quality data. The index of the reference genome was built using Bowtie v2.2.3 software and paired-end clean reads were aligned to the reference genome (ftp://ftp.ensembl.org/pub/release-97/fasta/oryctolagus_cuniculus/dna/Oryctolagus_cuniculus.OryCun2.0.dna.toplevel.fa.gz) using TopHat v2.0.14 software, and the mapped reads from each library were assembled with Cufflinks v2.2.1. The reference annotation-based transcript (RABT) assembly method in Cufflinks v2.2.1 was used to construct and identify mRNA transcripts from TopHat v2.0.14 alignment results. The model of fragments per kilobase of exon per million mapped reads (FPKMs) scores of genes in each sample was calculated with Cufflinks v2.2.1 (24), and heatmap was constructed with FPKM. Differentially-expressed genes (DEGs) between the CA (*n* = 3) and the NCA endometrial tissue (*n* = 3) were identified using DESeq2 R package (1.16.1) by screening for differential gene with a fold change ≥1 and an adjusted *P*-values < 0.05.

### Functional Enrichment and Interaction Analysis

Gene ontology (GO) enrichment analysis of DEGs was conducted using the GOseq R package, and DEGs were enriched into the significant overrepresentation of GO-biological process (GO-BP), molecular function (GO-MF) and cellular component (CC) terminologies. DEG enrichments were classified according to the Kyoto Encyclopedia of Gene and Genome (KEGG) algorithm using KOBAS 2.0 software. *P*-values were calculated using Benjamini-corrected modified Fisher's exact test. *P*-values < 0.05 were considered significant. The interaction network of the DEGs in the inflammatory mediator regulation of TRP channels, chemokine, focal adhesion and ECM-receptor interaction signaling pathways were constructed with online tool String 11.5 (https://cn.string-db.org/cgi/input?sessionId=inputgtsessionId&input_page_show_search=on).

### Quantitative Real-Time PCR

The purified RNA samples were reverse-transcribed using the PrimeScript® RT reagent kit with gDNA Eraser (Takara, Japan) following the manufacturer's protocol. Quantitative real-time PCR (RT-qPCR) was performed on a CFX 96 real-time PCR detection system (Bio-Rad, USA) in a 10 μl reaction mixture containing 3 μl dH_2_O, 5 μl 2 × SYBR premix EX Taq II, 0.5 μl of each forward and reverse primer and 1 μl cDNA (Supplementary Table 1). Following initial denaturation at 95°C for 3 min., PCR was carried out at 95°C for 10 s with an annealing temperature of 60°C for 30 s for 40 cycles. The β-actin gene was selected as a calibrator to normalize gene-specific RT-qPCR product expression. Finally, the relative expression levels of the genes were calculated using a relative quantification method (2^−Δ*ΔCt*^) (25).

### Immunostaining for Integrin-β and THBS

The immunohistochemical assay was performed as previously described, with modification (26). The NCA endometrial tissue was embedding in O.P.S compound for cryosectioning, and the placental tissue (the CA endometrium and chorio-allantoic membrane) was bedding in paraffin because placental tissue is more fragile than the NCA endometrium. After the sections were deparaffinized with xylene or rehydrated with gradient ethanol solution, the sections were rinsed in PBS, treated with proteinase-k, and incubated overnight with 5% goat serum (Boster Co., China), followed by incubation with anti-integrin-β polyclonal antibody (Cat. No. bs-0342R, Bioss Biotech. Inc., China) or anti-thrombospondin (THBS) polyclonal antibody (Cat. No. bs2715R, Bioss Biotech. Inc., China) diluted at 1:200 (v/v) in PBS, and washed with PBS for 15 min (5 min × 3). The immunoreaction products were identified with an SABC kit (Boster Co., China). Briefly, sections were incubated with biotinylated anti-mouse IgG+IgM+IgA (1:500), and avidin-peroxidase complex for 1 h each. Then, the immunoreaction products were visualized with a mixture consisting of 0.02% (w/v) 3, 3′-diaminobenzidine tetrahydrochloride and 0.005% (v/v) H_2_O_2_ in 50 mM Tris-HCl buffer (pH 7.6). The sections were counterstained with hematoxylin, dehydrated and covered. For negative control staining, the primary antibodies were replaced with normal mouse IgG (Boster Co., China).

### Enzyme Linked Immunosorbent Assay

After homogenization of the decidua and endometrial tissue, the total protein of each sample was extracted using a RIPA total protein extraction kit (Bioss, Biotech. Inc., China) and the concentration of total protein in each sample was adjusted to 2mg/mL. The levels of estrogen (E2), progesterone, interleukin-1beta (IL-1β), IL-6 and IL-8 were assessed using corresponding ELISA kit (Solarbio Life science Inc., China) according to the manufacturer's instructions.

## Results

### Summary of Transcriptome Data

To identify DEGs between the chorion-attached (CA) and non-chorion-attached (NCA) endometrium, transcriptome libraries were constructed using CA and NCA endometrial tissue, and a total of 147 million paired-end reads of 90 bp in length were generated. The total sequencing length was 44.17 Gb, representing approximately 15 × coverage of the whole rabbit genome. Among all clean reads, more than 77% of the reads were mapped to the reference genome (ftp://ftp.ensembl.org/pub/release-97/fasta/oryctolagus_cuniculus/dna/Oryctolagus_cuniculus.OryCun2.0.dna.toplevel.fa.gz) using TopHat v 2.0.14 (Table 1). The quality of this genome assembly is not high, and the genome of *Thorbecke* inbred and *New Zealand* white rabbits have large difference, which cause the low mapping percentage. Furthermore, 67.17-71.85 % of all reads were located within exons. If one transcript was expressed in all three biological replicates of the cDNA libraries, it was considered an expressed transcript for subsequent analysis. Consequently, 12,465 and 12,806 known transcripts were identified as being expressed in the CA and NCA endometrial tissues, respectively.

**Table 1:** Summary of transcriptome sequencing and alignment.

**Sample**	**CA1**	**NCA1**	**CA2**	**NCA2**	**CA3**	**NCA3**
Total raw reads (Raw base)	20,700,233 (6.21G)	25,808,529 (7.74)	26,644,963 (7.99G)	26,196,996 (7.86G)	23,051,170 (6.92G)	24,817,178 (7.45G)
Clean reads (Clean base)	20,357,987 (6.11)	25,462,894 (7.64G)	26,269,871 (7.88G)	25,809,208 (7.74G)	22,700,576 (6.81G)	24,347,133 (7.30G)
Q20 (%)	97.72	97.56	97.8	97.7	97.81	97.56
Valid ratio (%)	98.35	98.66	98.59	98.52	98.48	98.11
Total mapped ratio (%)	79.67	78.24	78.82	78.35	77.63	78.69
Unique mapped ratio (%)	79.39	77.95	78.34	77.95	77.35	78.43
Q20 (%)	97.72	97.56	97.8	97.7	97.81	97.56
GC content (%)	54.56	54.02	54.42	53.87	56.35	55.06

*CA1, CA2, and CA3 are chorion-attached endometrium, and NCA1, NCA 2, and NCA 3 are non-chorion-attached endometrial tissues of the corresponding individuals, CA1, CA2, and CA3*.

**Figure 1:**
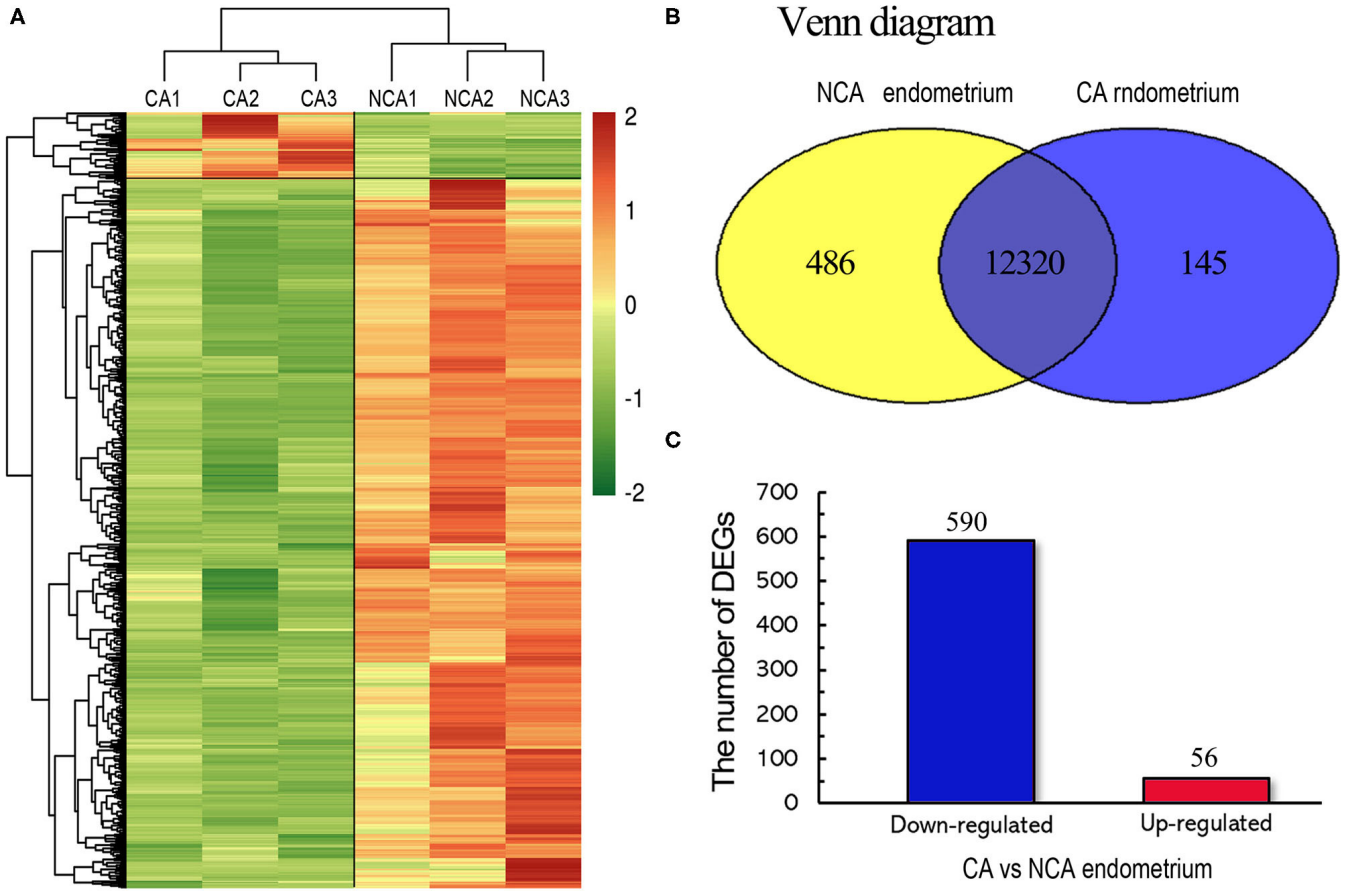
Genome-wide distribution between the chorion-attached (CA) and non-chorion-attached (NCA) endometrium of rabbit. **(A)** Heatmap diagram of DEGs and hierarchical clustering of samples. **(B)** Venn diagram of expressed known genes in the chorion-attached and non-chorion-attached endometrium. **(C)** The number of up- and down-regulated DEGs. CA1, CA2, and CA3 is the chorion-attached endometrial tissue, and NCA1, NCA2, and NCA3 is the non-chorion-attached endometrial tissue of the corresponding individual, CA1, CA2, and CA3 (*n* = 3).

To assess the reproducibility and reliability of the transcriptome libraries, Pearson's correlations of transcriptional level of the total expressed genes from all samples were analyzed. Three biological replicates of each group had high correlation (*r* = 0.82 in the CA group, *r* = 0.87 in the NCA group), which was higher than the paired Pearson's correlation coefficient (*r* = 0.7232, the average correlation coefficient of CA1-NCA1, CA2 -NCA2 and CA3-NCA3), and the non-paired Pearson's correlation coefficient (*r* = 0.5749). The results verified the high reproducibility and reliability. The heatmap of the hierarchical clustering showed that the DEGs data were reproducible (Figure 1A). The validity of transcriptome sequencing was assessed using qRT-PCR of six genes (TLR4, TGFα, WNT3, LAMB2, PPET2 and IL23α), and the expression level of these genes have significant difference between CA and NCA endometrium. The six genes also involved in immune regulation and cell adhesion. The results indicated that the expression patterns of these genes were highly consistent between the RNA-seq and qRT-PCR (Pearson's *r* = 0.765, *p* < 0.01; Supplementary Figure 2). The global transcriptome was presented in the Venn diagram (Figure 1B), we identified 12,951 known genes, and among these, 12,320 known genes were co-expressed in the CA and NCA endometrium. The results showed that many co-expressed genes were expressed in the CA and NCA endometrium, and a total of 646 DEGs was identified in the CA endometrium compared with the NCA endometrium; 590 DEGs were down-regulated and 56 DEGs were up-regulated (Figure 1C).

### Top 20 Up and Down-Regulated Differentially Expressed Genes

A total of 646 DEGs between the chorion-attached (CA) and non-chorion-attached (NCA) endometrium were identified, and the top 20 down- and up-regulated genes in the CA endometrium are presented in Tables 2, 3, respectively. *PLCL1, ADRB3, PTGIS, SLC26A10, SPEG, TAGAP, TCF23, ADAM33, MYLK, MG72, JP1, FAT4, SLC2A4, LIMS2, KCNH2, CD46, SEPT6, SLC24A2, CARD6, SMOC2* and *REEP1* were highly expressed in the NCA endometrium (Table 2) compared to the CA endometrium (log2FC =-5.644 — −4.266; FDR p < 0.05), while these genes, *PADI2, IL23A, TP63, IL20, WNT-11, EMB, FAM78A, HMGCS2, FAM65B, GJD4, CX3CR1, RTN4RL2, PHYHIP, RASL11A, CA2, DUSP10, ECEL1, PFKFB3, ARNTL2* and *CIART* were highly expressed in the CA endometrium (Table 3) compared to the NCA endometrium (log2FC = 2.475— 4.637; FDR *p* < 0.05).

**Table 2:** Top 20 down-regulated DEGs between the chorion-attached (CA) and non-chorion-attached (NCA) endometrium.

**Gene symbol**	**Full name**	***P*-value**	**FDR**	**log_**2**_FC**
*PLCL1*	Phospholipase C-like 1	4.5488E-18	9.99E-15	−5.644
*ADRB3*	Adrenoceptor-beta3	1.1556E-13	6.62E−11	−5.618
*PTGIS*	Prostaglandin I 2 synthases	1.012E-13	6.06E-11	−5.122
*SLC26A10*	Solute carrier family 26, member 10	4.6549E-15	5.22E-12	−4.777
*SPEG*	Striated muscle preferentially expressed protein kinase	3.4472E-10	6.68E-08	−4.703
*TAGAP*	T-cell activation Rho GTPase activating protein	1.9626E-24	1.29E-20	−4.693
*TCF23*	Transcription factor 23	2.4728E-14	1.92E-11	−4.563
*ADAM33*	ADAM metallopeptidase domain 33	1.7502E-12	7.69E-10	−4.532
*MYLK*	Myosin light chain kinase	8.0753E-10	1.4E-07	−4.529
*MG72/JP1*	Junctophilin-1	5.7588E-09	8.07E-07	−4.519
*FAT4*	FAT atypical cadherin 4	3.4229E-13	1.8E-10	−4.496
*SLC2A4*	Solute carrier family 2 (glucose transporter)	9.4332E-08	9.14E-06	−4.456
*LIMS2*	LIM and senescent cell antigen-like domains 2	4.2557E-11	1.17E-08	−4.413
*KCNH2*	Potassium voltage-gated channel subfamily H member 2	2.2876E-09	3.57E-07	−4.386
*CD46*	CD46 molecule, complement regulatory protein	8.9324E-07	5.94E-05	−4.370
*SEPT6*	Septin 6 (septin GTPase family)	1.5841E-10	3.48E-08	−4.332
*SLC24A2*	Solute carrier family 24 (sodium/potassium/calcium exchanger)	2.8723E-18	7.57E-15	−4.326
*CARD6*	Caspase recruitment domain family, member 6	6.3969E-10	1.17E-07	−4.302
*SMOC2*	SPARC related modular calcium binding 2	1.1588E-15	1.61E-12	−4.267
*REEP1*	Receptor accessory protein 1	1.5283E-10	3.41E-08	−4.266

**Table 3:** Top 20 up-regulated DEGs between the CA and NCA endometrium.

**Gene symbol**	**Full name**	***P-*value**	**FDR**	**log2FC**
*PADI2*	Peptidyl arginine deiminase, type II	1.3062E-07	1.19E-05	4.637
*IL23A*	Interleukin 23, alpha subunit	1.0314E-05	0.000441	3.977
*TP63*	Tumor protein p63	3.5924E-05	0.001243	3.839
*IL20*	Interleukin 20	5.0592E-05	0.001667	3.730
*WNT-11*	Protein Wnt	6.8296E-05	0.00206	3.719
*EMB*	Embigin	8.9132E-05	0.002585	3.519
*FAM78A*	Family with sequence similarity 78, member A	1.7201E-05	0.000702	3.518
*HMGCS2*	3-hydroxy-3-methylglutaryl coenzyme A synthase	0.00041207	0.008647	3.299
*FAM65B*	Family with sequence similarity 65, member B	0.00010128	0.002852	3.297
*GJD4*	Gap junction protein	0.00021237	0.005107	3.293
*CX3CR1*	CX3C chemokine receptor 1	0.00013019	0.003483	3.208
*RTN4RL2*	Reticulon 4 receptor-like 2	5.6258E-05	0.001804	3.079
*PHYHIP*	Phytanoyl-CoA 2-hydroxylase interacting protein	0.00044586	0.009195	3.050
*RASL11A*	RAS-like, family 11, member A	1.9642E-05	0.00077	2.988
*CA2*	Carbonic anhydrase 2	0.0003132	0.006972	2.880
*DUSP10*	Dual specificity phosphatase 10	0.00034109	0.007504	2.788
*ECEL1*	Endothelin converting enzyme-like 1	0.00028101	0.006357	2.749
*PFKFB3*	6-phosphofructo-2-kinase/fructose-2,6- bisphosphatase 3	0.00010232	0.002875	2.701
*ARNTL2*	Aryl hydrocarbon receptor nuclear translocator-like 2	0.00019704	0.004844	2.553
*CIART*	Circadian associated repressor of transcription	0.00037716	0.008095	2.475

### Functional Enrichment Analysis of Differentially Expressed Genes (DEGs)

**Figure 2:**
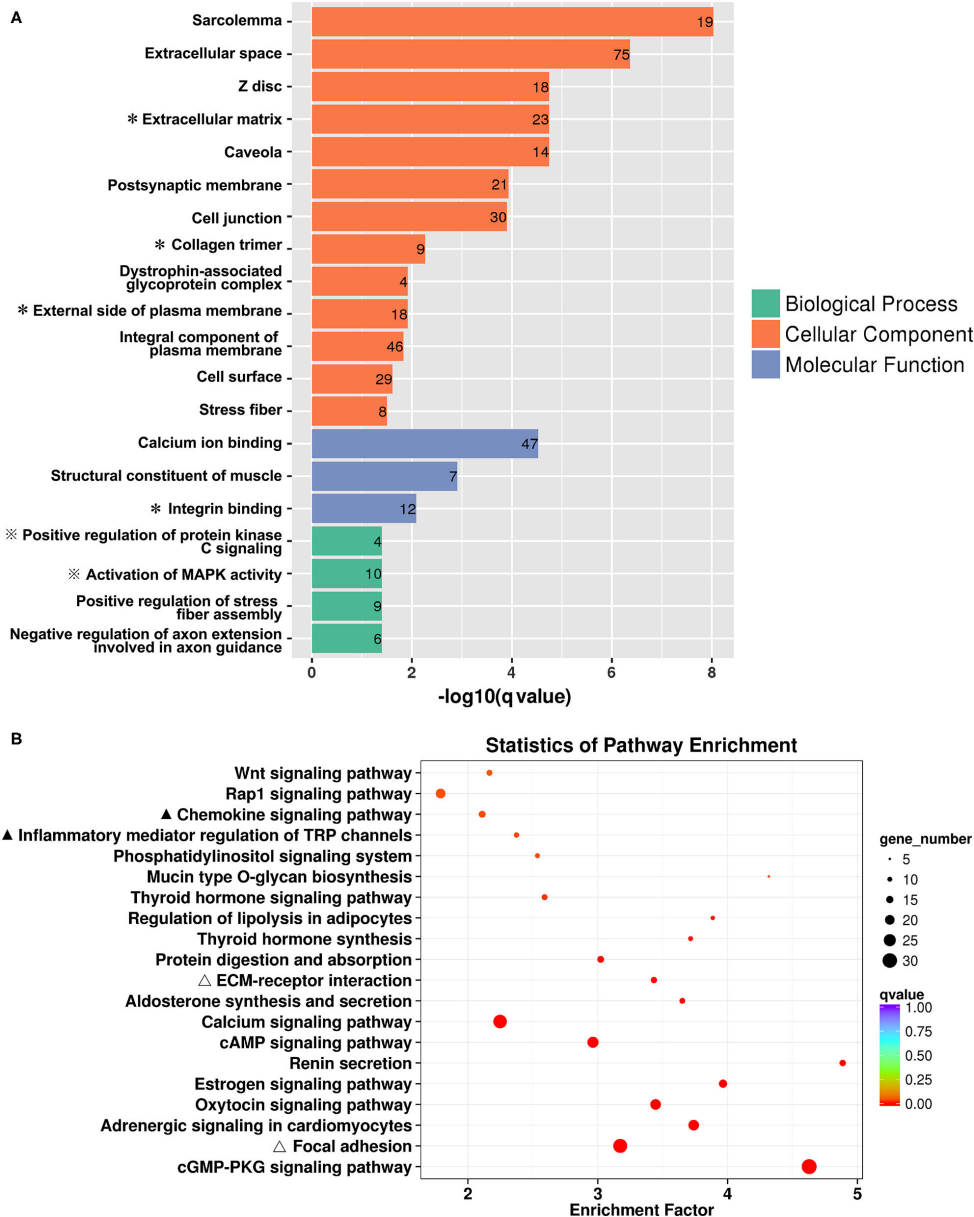
Functional enrichment of DEGs between the chorion-attached (CA) and non-chorion-attached (NCA) endometrium. **(A)** Gene ontology (GO) categories enriched for DEGs, **(B)** KEGG pathway enrichment of these DEGs. The q values were calculated using a Benjamini-corrected modified Fisher's exact test. A * next to the name indicated the GO terms that were related to ECM-receptor and cell adhesion, and ※ indicated the GO terms that were related to immunoregulation. A ▴ indicated the KEGG pathways involved in immunoregulation, and a Δ indicated the KEGG pathways that were involved in ECM-receptor interaction and cell adhesion.

The 646 DEGs were subjected to GO enrichment analysis. As shown in Figure 2A, the most significantly over-represented GO terms were related to the biological function of cellular or extracellular matrix interaction (extracellular space, extracellular matrix, cell junction and cell surface); the molecular function of protein kinase regulation (positive regulation of protein kinase C signaling, activation of MAPK activity); and the regulation of fiber or axon formation (positive regulation of stress fiber assembly, negative regulation of axon extension involved in guidance). KEGG analysis (Figure 2B) showed that the DEGs between CA and NCA endometrium were mainly involved in immune regulation (chemokine signaling pathways, inflammatory mediator regulation of TRP channels), reproductive hormone regulation (estrogen signaling pathway, oxytocin signaling pathway) and cellular adhesions (focal adhesion, ECM-receptor interaction). The transcriptome data strongly indicated that immune regulation and cell adhesion were the main biological functions in the mid-gestational CA and NCA endometrium.

### Differential Expression of Genes Related to Inflammatory Mediator Regulation and Chemokine Signaling Pathways

**Figure 3:**
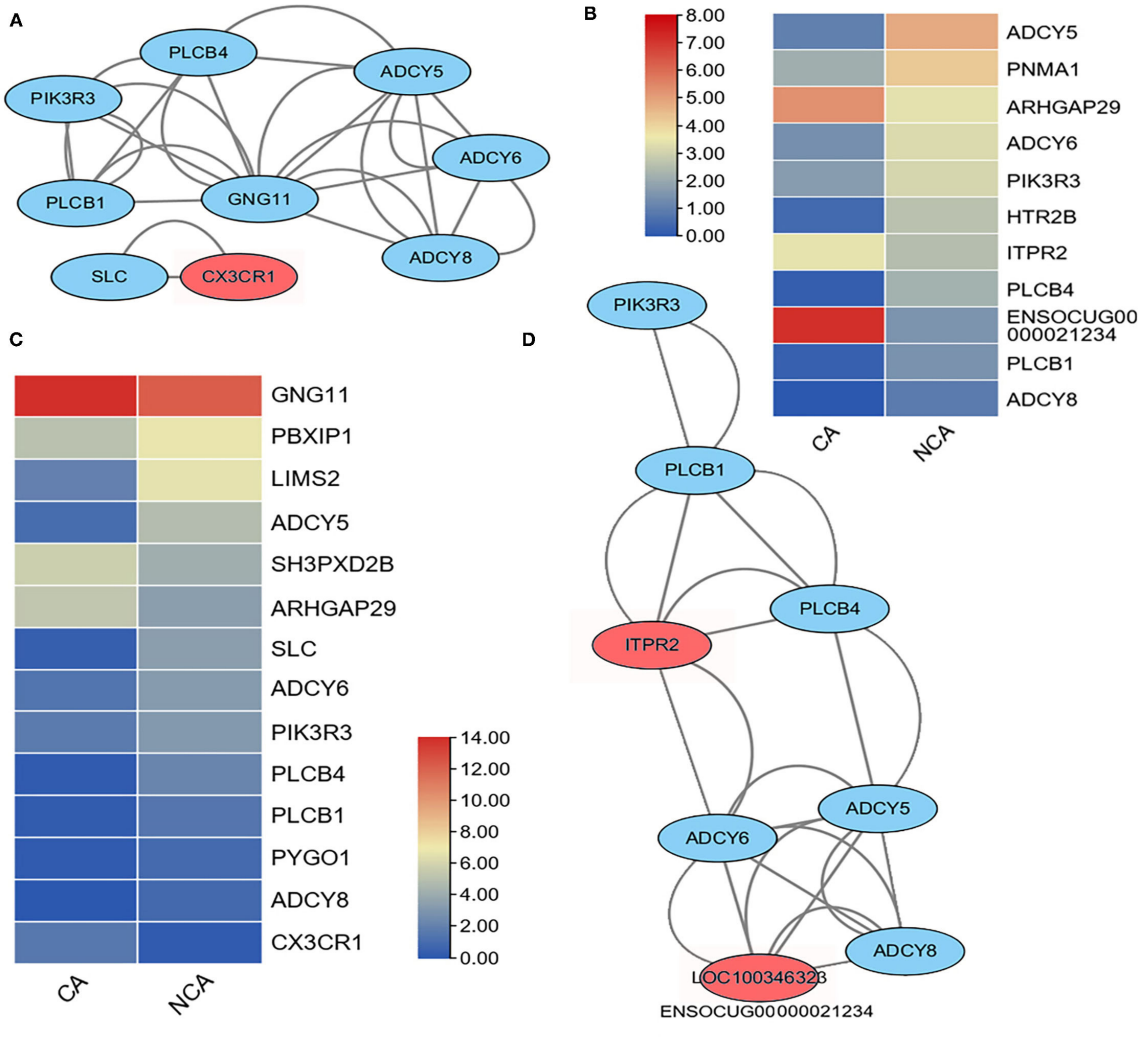
Heatmap and interaction network of differentially expressed genes related to inflammatory mediator regulation of TRP channels and chemokine signaling pathways. **(A)** interaction network and **(C)** expression heatmap of DEGs related to chemokine signaling pathway. **(D)** interaction network and **(B)** expressive heatmap of DEGs related to inflammatory mediator regulation of TRP channels. The expression level of genes between the chorion-attached endometrium (CA) and the homologous non-chorion-attached endometrium (NCA) were compared using paired *t*-test using PFKM of each DEG in the pathway. The interaction network of the DEGs in the inflammatory mediator regulation of TRP channels and chemokine signaling pathways were constructed with online tool String 11.5.

The maintenance of pregnancy requires proper physiological regulation of immune system function (16–18), and the KEGG analysis of DEGs indicated the up-regulation of genes involved in inflammatory mediator regulation of TRP channel and chemokine signaling pathways in the CA and NCA endometrium. Therefore, these inflammatory mediator regulatory genes in the TRP channel and chemokine signaling pathways were analyzed. A total of 148 genes related to inflammatory mediator regulation of TRP channels were expressed in CA and NCA endometrium, of which 11 had significant differences in expression between CA and NCA. 210 genes related to the chemokine signaling pathway were detected in CA and NCA endometrium, of which 14 had significant differences in expression between CA and NCA. This list of DEGs involved in inflammatory mediator regulation of TRP channels and chemokine signaling pathways was used to construct an interaction network and expression heatmap (Figure 3). Among the 14 DEGs related to chemokine signaling pathways, nine of them were assigned to two interaction networks (Figure 3A). The expression of *CX3CR1* and *GNG11* in the CA was greater than in the NCA endometrium, and the other 12 DEGs in the CA were lower than in the NCA endometrium (Figure 3C). Among the 11 DEGs related to inflammatory mediator regulation of the TRP channel, the expression of ARHGAP29, ENSCOCUG00000021234 (LOC100246323) in the CA was greater than in the NCA endometrium, and the other 9 DEGs in the CA were lower than in the NCA endometrium (Figure 3B). Nine of them were assigned to an interaction network (Figure 3D). Moreover, the interaction networks of DEGs in inflammatory mediator regulation of TRP channel and chemokine signaling pathways have six of the same hub-genes (*PLCB1, PLCB4, PIK3R3, ADCY5, 6* and *8*). These results suggested that the hub-genes related to immunoregulation had a lower expression level in the CA endometrium compared to the NCA endometrium.

### Differential Expression of Genes Related to Focal Adhesion and ECM-Receptor Interaction

**Figure 4:**
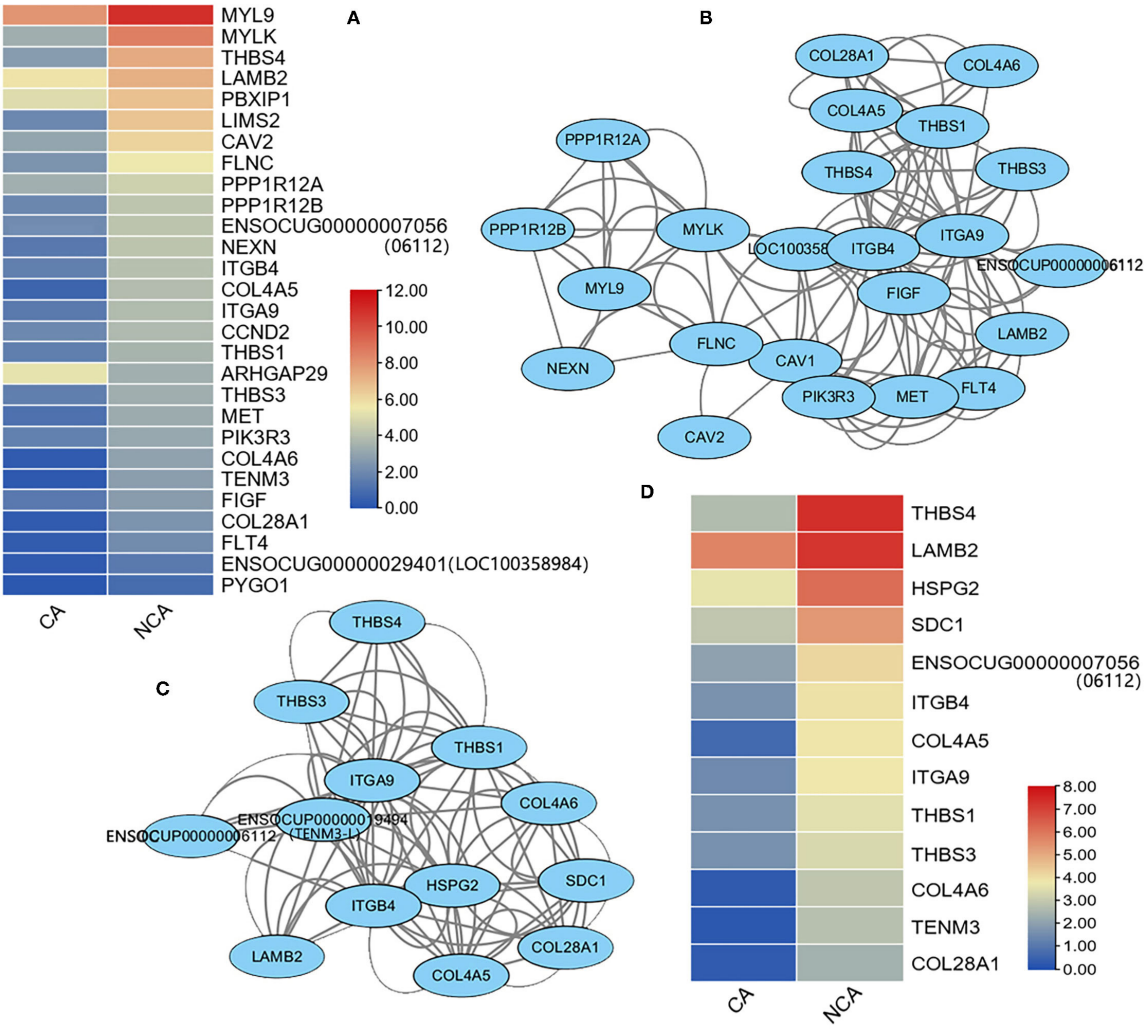
Heatmap and interaction network of differentially-expressed genes related to focal adhesion and ECM-receptor interaction signaling pathways. **(A)** Expression heatmap, **(B)** interaction network of DEGs related to focal adhesion, **(C)** interaction network, and **(D)** expression heatmap of DEGs related to ECM-receptor interaction. The expression level of genes between the chorion-attached endometrium (CA) and the homologous non-chorion-attached endometrium (NCA) were compared using paired *t*-test according PFKM of each DEG in the pathway. The interaction network of the DEGs in focal adhesion and ECM-receptor interaction signaling pathways were constructed with online tool String 11.5.

The KEGG analysis of the current study revealed the DEGs that were enriched in the function of focal adhesion and ECM-receptor interaction, while embryo attachment and implantation and pregnancy maintenance were related to the extracellular matrix (ECM) and focal adhesion (13–15). These genes were analyzed and 28 DEGs in the CA vs. NCA were found among the 215 observed genes related to focal adhesion, and only the expression level of the ARHGAP29 gene in the CA endometrium was significantly higher than that in the NCA endometrium (Figure 4A). An interaction network was constructed from the 23 DEGs related to focal adhesion (Figure 4B). Among the 86 observed genes related to ECM-receptor interaction, 13 DEGs of CA vs. NCA were used to construct an interaction network (Figure 4C). The expression level of these 13 genes in the NCA endometrium was significantly higher than that in the CA endometrium (Figure 4D). The interaction networks of DEGs in focal adhesion and ECM-receptor interaction pathway shared the same 10 hub-genes (*THBS1, 3*, and *4, ITGB4, ITGA9, COL4A5* and *6, COL28A1, LAMB2, ENSOCUG00000006112*). These results suggested that the genes related to focal adhesion and ECM-receptor interaction had a higher expression level in the NCA endometrium than in the CA endometrium, and THBS, collagen (COL), laminin (LAMB), and integrin (ITG) were hub-genes in the focal adhesion and ECM-receptor interaction pathway.

### Concentration of IL-1β, IL-6 and IL-8 and Localization of Integrin-β and THBS in the NCA Endometrium

**Figure 5:**
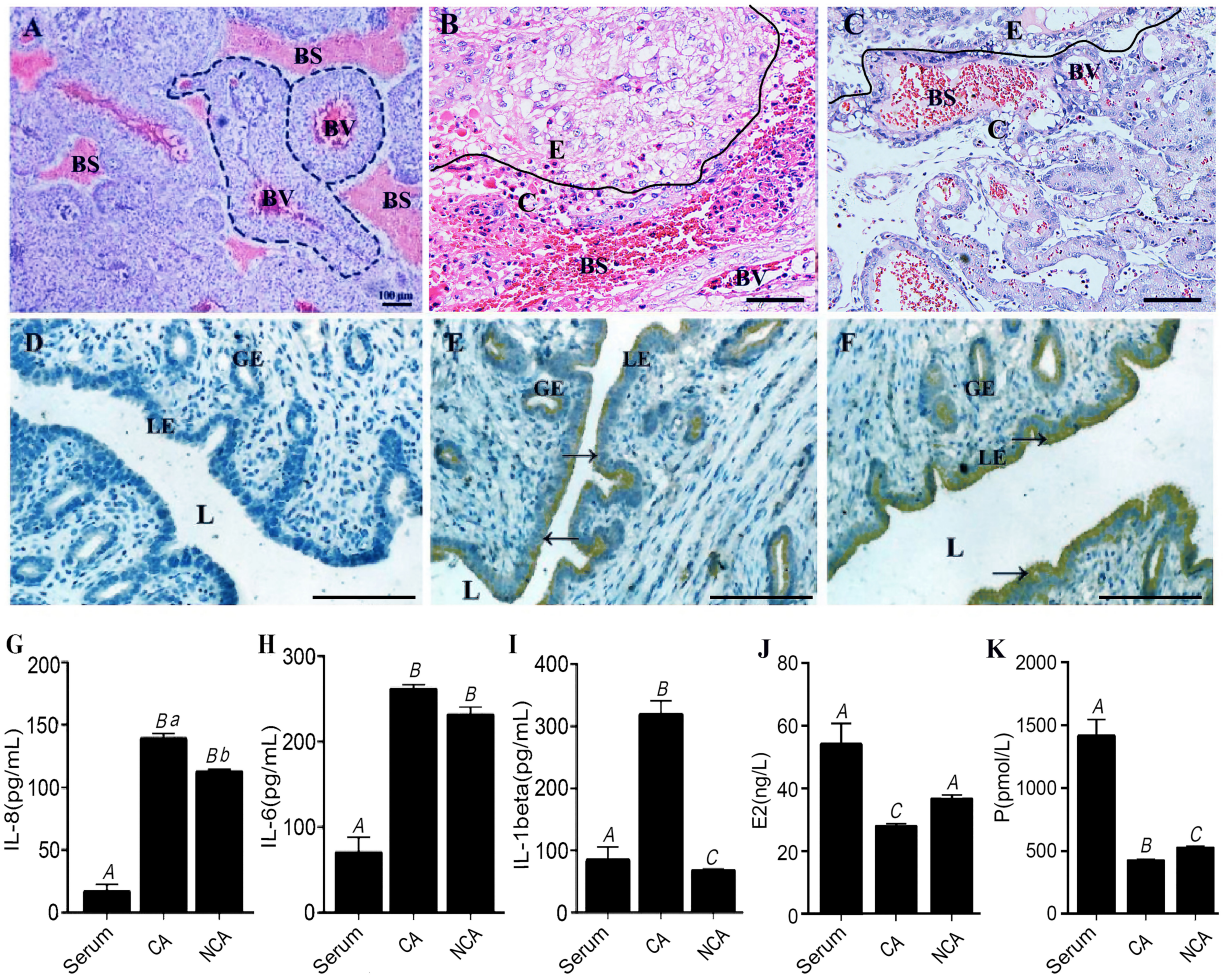
Concentration of IL-1β, IL-6 and IL-8 and localization of integrin-β and THBS in the CA and NCA endometrium. **(A,D)** are the negative controls for the placenta and endometrium in the immunohistochemical (IHC) assay. Integrin wasn't present in the placenta **(B)**, but was localized in the NCA endometrium **(E)**. THBS1 wasn't present in the placenta **(C)**, but was localized in the NCA endometrium **(F)**. Dotted line in this figure **(A)** indicated the placental villi in blood sinus of maternal endometrial layer. BV, blood vessels in the placental villi; BS, blood sinus of maternal endometrial layer; E, CA endometrium; C, chorion; L, lumen; LE, luminal epithelium; GE, glandular epithelium. Scale bar in **(A–F)** is 100 μm. **(G–K)** respectively indicated the concentration of IL-8, IL-6, IL-1β, Estradiol (E2) and Progesterone (P) in the serum, CA and NCA endometrium. The concentration was compared turkey's test, and different capital letters meant *p* < 0.01, while different lowercase letters meant *p* < 0.05. → Positive cells by IHC. CA, chorion-attached endometrium; NCA, non-chorion-attached endometrium.

Integrin-β and THBS were localized in the CA and NCA endometrium by immunohistochemical (IHC) assay on paraffin-section or cryosection slides (Figures 5A–F). Integrin-β and THBS1 at high abundance were localized in the luminal epithelium and the glandular epithelium of the NCA endometrium. Conversely, integrin-β and THBS1 were not detected in CA endometrial cells at the maternal-fetal interface. To ascertain the immune situation in the CA and NCA endometrium, the concentration of IL-8, IL-6 and IL-1β were measured in the serum, the CA and the NCA endometrium (Figures 5G–I) using enzyme-linked-immunosorbent serologic assay (ELISA), The concentrations of IL6 and IL-8 in the CA and NCA endometrium were significantly much higher than in the sera (*p* < 0.05), and the concentration of IL-1β in the CA endometrium was significantly greater than that in the sera (*p* < 0.05). These data suggest that chemokines and pro-inflammatory cytokines accumulated at the maternal-fetal interface. In addition, the concentration of estradiol (E2) and progesterone (P) in serum was greater than that in the CA endometrium (*p* < 0.05), and the concentration of P in serum was greater than that in the NCA endometrium (*p* < 0.05) (Figures 5J,K).

## Discussion

Our study has revealed a difference in gene expression between the chorion-attached (CA) and non-chorion-attached (NCA) endometrium of mid-gestation in rabbit. (1) 646 DEGs between the CA and NCA endometrium were identified, and (2) the DEGs were mainly involved in immune regulation, reproductive hormone regulation and cellular adhesion. (3) The hub-genes related to inflammatory mediator regulation of TRP channel and chemokine signaling pathway, and related to focal adhesion and the ECM-receptor interaction signaling pathway in NCA endometrium were significantly higher than in CA endometrium. (4) IL-6 and IL-8 in the endometrium were significantly much higher than in the sera at mid-gestation.

In our study, transcription in the NCA endometrium was more active than that in the CA endometrium. The top 20 down- and up-regulated DEGs in the CA endometrium have been identified in several studies. The *SLC2A4, SLC24A2, SLC26A10, PLC1, PTGIS, MYLK, IL23A, WNT3*, and *WNT11* genes in the maternal placenta (the chorion-attached endometrium) have been detected in the placenta-attached decidua of horses (27), rats (28), humans (22) and hamsters (29). The solute carrier (SLC) superfamily is the largest group of membrane transporters, comprising 65 families (SLC1-65) with more than 400 identified genes, and *SLC2A4* is in the glucose/glucosamine transporter family (30). Previous studies revealed that SLC2 is the most abundant glucose transporter in human endometrium and is up-regulated during decidualization (31). *SLC24A2* and *SLC26A10* are members of the Na+/Ca2+-K+ exchanger family and multifunctional anion exchanger family, respectively (32). *TAGAP, LIMS2, IL23A, IL20*, and *CX3CR1* are involved in immune regulation of the cross-talk between decidual stromal cells, trophoblast cells, and immune cells during pregnancy (33). *GJD4, IL23A, WNT11, CX3CR1, TP63*, and *IL20* regulate multicellular organismal processes and responses to stimuli (34–38). ADRB3, PLCL1, and PTGIS are involved in biological regulation and responses to stimuli (39–42).

Previous studies indicated that the PKC and MAPK signaling pathways are widely involved in vascular remodeling, trophoblast invasion, and intracellular signal pathways in the process of placental development in the decidualized endometrial layer (43–45). Pregnancy results in an immuno-suppressed state that is necessary for the maternal immune system to tolerate the developing fetus (46). During pregnancy, the maternal immune system adapts to permit tolerance of the fetal allograft while maintaining a defense against harmful pathogens (47, 48). The innate immune system plays an essential role and maintains an appropriate environment for the developing fetus while still protecting the health of the mother (46). The mid-gestational placenta supports proportional fetal growth, organ development and fine-scale differentiation, as well as continuing maternal adaptation to pregnancy (22). A successful pregnancy requires a fine-tuned and highly regulated equilibrium between immune activation and embryonic antigen tolerance (49). Activation of the ECM receptors and interaction with the PI3K-Akt signaling pathway are involved in maintaining maternal tolerance (50). Focal adhesions are macromolecular complexes comprised of heterodimeric transmembrane integrin receptors that regulate ECM receptors on the luminal epithelium of the endometrium (51, 52). It has been reported that focal adhesions play an important role at the maternal-fetal interface in many species (51). Our study also confirmed involvement of the endometrium in this physiological function in mid-gestation.

The maintenance of pregnancy requires proper physiological regulation of immune system function (16–18), and hub-genes related to immunoregulation had a lower expression level in the CA endometrium compared to the NCA endometrium in the present study. Some studies have demonstrated that a variety of chemokines were expressed in the endometrium and decidua in early pregnancy (53–58), and a successful pregnancy depends on the spatiotemporal expression of chemokines and signal pathway members in different stages of pregnancy to achieve the decidualization of uterine luminal epithelium, trophoblast adhesion, embryo implantation and immune tolerance (59, 60). Messenger RNA expression of the chemokines, CCL2, CCL4, CCL5, CCL8, CXCL2, CXCL8, CXCL10 and CXCL12, and both the mRNA and protein of their receptors, CCR1, CCR2, CCR3, CCR5, CXCR2, CXCR3, and CXCR4, were detected in porcine luminal epithelial cells (53). CXCL12 and CXCR4 were expressed at the maternal-fetal interface of mouse, and CXCL12 increased migration of regulatory T cells into the uterus during pregnancy (61). Our results revealed that the genes related to inflammatory mediator regulation of the TRP channel and chemokine signaling pathways were expressed in the CA and NCA endometrium, and their expression in NCA endometrium was higher than in CA endometrium. The results were consistent with previous results showing that placenta has the function of immune escape (16–18). Embryo implantation evolved an ancestral inflammatory attachment reaction (60) and immune cytokines and cells in the decidua and endometrium maintain intrinsic inflammatory responses and immune modulating secretion (33, 55, 60).

The interaction of ECM and integrin is widely involved in intercellular adhesion, crosstalk and communication (62), and involved in embryo implantation in the endometrium in the proliferative phase and the decidualized endometrium of rat (63), sheep (64), dog (65), human (66), goat (67) and mouse (68). The THBS family, THBS1, THBS3, and THBS4, play key roles in the success of pregnancy recognition, implantation and maintenance (69). Integrins are glycoprotein transmembrane receptors that exist as heterodimers composed of non-covalently linked alpha and beta subunits, and they are critical for the formation of focal adhesions, cell migration, proliferation and development of the actin cytoskeleton. Integrins regulate adhesion at the fetal-maternal interface by interacting with secreted phosphoprotein 1 and fibronectin (FN) (70). The thrombospondins are ECM proteins belonging to the THBS family consisting of five members (71), and they can interact with various ECM proteins, cytokines, receptors, and growth factors (71, 72). The ECM-integrin ligand plays a key role in the process of embryo adhesion at the maternal-fetal interface. Our study revealed a novel finding that the ECM-integrin ligands were highly expressed in the NCA endometrium, but not in the CA endometrium at mid-gestation. Previous studies confirmed that some chemokines from the placenta could regulate ECM and adhesion molecules (73). According to our data, the CA and NCA endometria released chemokines in mid-gestation that might up-regulate ECM-integrins in the NCA endometrium; then, uterine luminal epithelial adhesions could build a barrier of immune tolerance and defense around the conceptus.

The current study suggests that our understanding of the mechanism of the immune tolerance of the CA and NCA endometrium, makes it likely that the chemokines from the maternal-fetal interface induce expression of ECM-integrins in the adjacent endometrium to build an adhesive cellular barrier.

## Conclusions

Based on the transcriptomes of the CA and NCA endometrium at the mid-stage of pregnancy, we found differences in the gene expressive profile between the CA and NCA endometrium of rabbit. In particular, transcription in the NCA endometrium was more active than that in the CA endometrium, and the hub-genes related to inflammatory mediator regulation of TRP channels, chemokine signaling, focal adhesion and ECM-receptor interaction signaling pathways in the NCA endometrium were significantly higher than those in the CA endometrium. The results provide the reference for understanding the CA endometrium mediates maternal and fetal immune tolerance, and the involvement of the NCA endometrium in cell adhesion. However, there are few studies on the gene expression profiles of CA and NCA endometrium, and some placental membrane cells maybe invade in the CA endometrium. Therefore, the spatial transcriptome of placenta and endometrium will provide us with a more complete picture of the expression profile of the associated genes and their function in the CA and NCA endometrium.

## Data Availability Statement

The datasets presented in this study can be found in online repositories. The names of the repository/repositories and accession number(s) can be found below: https://www.ncbi.nlm.nih.gov/geo/, GSE152905.

## Ethics Statement

The animal study was reviewed and approved by Animal Ethics Monitoring Committee of Sichuan Agricultural University and Sichuan Animal Science Academy (Appr. No. SASA201905).

## Author Contributions

XM and MZ: conceptualization. LX and YZ: data curation and writing the original draft. XM: funding acquisition. LK, CL, and ZG: resources. MY, XX, and DH: methodology. MY, CL, YR, and ZW: investigation. MZ: reviewing and editing the draft. All authors contributed to the article and approved the submitted version.

## Funding

This work was supported by the Fundamental Research Funds for the Sichuan Province Institute of Animal Husbandry Research (SASA201905).

## Conflict of Interest

The authors declare that the research was conducted in the absence of any commercial or financial relationships that could be construed as a potential conflict of interest.

## Publisher's Note

All claims expressed in this article are solely those of the authors and do not necessarily represent those of their affiliated organizations, or those of the publisher, the editors and the reviewers. Any product that may be evaluated in this article, or claim that may be made by its manufacturer, is not guaranteed or endorsed by the publisher.
