# Supplementary material for: Transcriptome Comparison of Chorion-Attached and Non-chorion-attached Endometrium in Mid-gestation of Rabbit
Source: Front Vet Sci. 2022 Mar 10;9:838802. doi: 10.3389/fvets.2022.838802 (PMC8965606; doi:10.3389/fvets.2022.838802)
Supplement: Supplementary file 1 [file Data_Sheet_1.docx]

**Transcriptome comparison of chorion-attached and non-chorion-attached endometrium in mid-gestation**


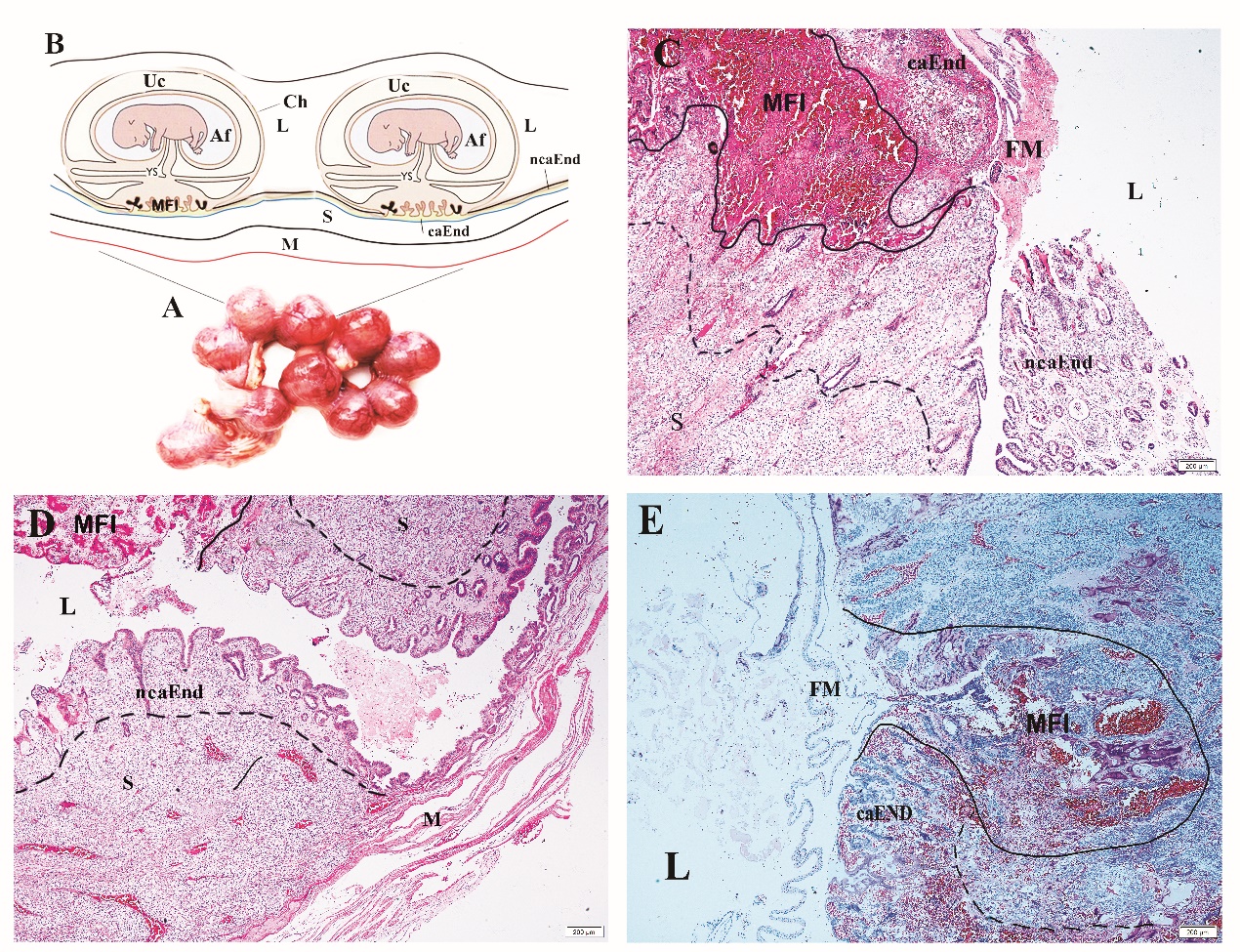


**Supplementary Figure S1** **Sampling diagram and sample evaluation**. (**A**) The uterine body with ten fetuses in mid-gestation. (**B**) Sample diagram: caEnd indicated the chorion-attached endometrium (the decidua), ncaEnd indicated the non-chorion-attached endometrium (endometrium) (Ch: chorion; S: stromal layer of endometrium; M: myometrium of uterine wall; Af: amniotic fluid cavity; L: uterine lumen; YS: Yolk sac). (**C**)(D)(E) Hematoxylin-eosin staining of placental cryo-sections for three sample evaluation (MFI: maternal-fetal interface; FM: membrane; S: stromal layer of endometrium. Scale bar = 200 μm).

**Supplementary Table S1.** Primer information and annealing temperatures for qRT-PCR.

| **Gene**  **symbol** | **Primer** | **Ta(℃)** | **Product**  **length** | **Accession No.** |
| --- | --- | --- | --- | --- |
| *β-actin* | F: 5’-GGAGATCGTGCGGGACAT-3’ | 60°C | 223 bp | NM_001101683.1 |
|  | R: 5’-GTTGAAGGTGGTCTCGTGGAT-3 |  |  |  |
| *TLR4* | F: 5’-GAGCACCTGGACCTTTCAAATAAC-3 | 60°C | 235 bp | NM 001082732 |
|  | R: 5’-GAACTTCTAAACCACTCAGCCCTTG-3 |  |  |  |
| *IL23A* | F: 5’-CGTTGCATCAGGGAGGTGAT-3 | 60°C | 103 bp | XM_002711079.3 |
|  | R: 5’-GGTAGCTGAGAATGCACGGT-3 |  |  |  |
| *TGFA* | F: 5’-CTCTGTCTGCTCGGGTTGTT-3 | 60°C | 132 bp | XM_008254277.2 |
|  | R: 5’-TGGACTTGGACGCTGATGAC-3- |  |  |  |
| *WNT3* | F: 5’-TTGAGTCTTTCGCGGTGCTC-3 | 60°C | 123 bp | XM_008271640.2 |
|  | R: 5’-CTAAGGCAGCTTTGATGGGC-3 |  |  |  |
| *LAMB2* | F: 5’-AATCCTGTCTGCCTTGAGCC-3 | 60°C | 107 bp | XM_002713417.2 |
|  | R: 5’-GTCACCCCCGCTAAACATCT-3 |  |  |  |
| *PPET2(END2)* | F: 5’-CTGTGCCTCACCTGTCTGTA-3 | 60°C | 173 bp | XM_008263945.2 |
|  | R: 5’-TTCCCCTGGTCAGCCAATTC-3 |  |  |  |

Ta: Annealing temperature.


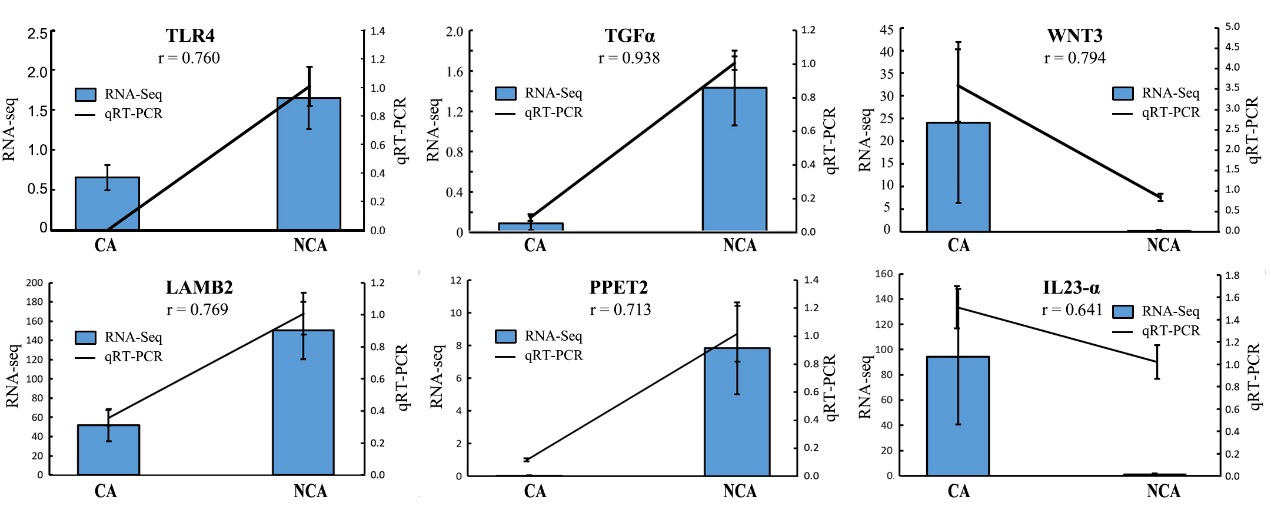


**Supplementary Figure S2** Validation of DEGs by qPCR. The *r* value represents the Pearson correlation coefficient between two methods. CA=chorion-attached endometrium, NCA=non-chorion-attached endometrium.
